# Modeling of levothyroxine in newborns and infants with congenital hypothyroidism: challenges and opportunities of a rare disease multi-center study

**DOI:** 10.1007/s10928-021-09765-w

**Published:** 2021-06-11

**Authors:** Gilbert Koch, Britta Steffens, Stephanie Leroux, Verena Gotta, Johannes Schropp, Pascal Gächter, Freya Bachmann, Tatjana Welzel, Marco Janner, Dagmar L’Allemand, Daniel Konrad, Gabor Szinnai, Marc Pfister

**Affiliations:** 1 Pediatric Pharmacology and Pharmacometrics, University Children’s Hospital Basel UKBB, University of Basel, Basel, Switzerland; 2 Department of Mathematics and Statistics, University of Konstanz, Konstanz, Germany; 3 Pediatric Endocrinology, Diabetology and Metabolism, Department of Pediatrics, Bern University Hospital , University of Bern, Bern, Switzerland; 4 Department of Pediatric Endocrinology and Diabetology, Children’s Hospital of Eastern Switzerland, St. Gallen, Switzerland; 5 Division of Pediatric Endocrinology and Diabetology and Children’s Research Centre, University Children’s Hospital, Zurich, Switzerland; 6 Pediatric Endocrinology and Diabetology, University Children’s Hospital Basel UKBB, University of Basel, Basel, Switzerland; 7 Department of Clinical Research, University of Basel and University Hospital Basel, Basel, Switzerland

**Keywords:** Normalization, Reference range, Scale/location-scale, Pediatrics, Pharmacokinetics, Levothyroxine, Congenital hypothyroidism, Rare disease, Thyroid

## Abstract

Modeling of retrospectively collected multi-center data of a rare disease in pediatrics is challenging because laboratory data can stem from several decades measured with different assays. Here we present a retrospective pharmacometrics (PMX) based data analysis of the rare disease congenital hypothyroidism (CH) in newborns and infants. Our overall aim is to develop a model that can be applied to optimize dosing in this pediatric patient population since suboptimal treatment of CH during the first 2 years of life is associated with a reduced intelligence quotient between 10 and 14 years. The first goal is to describe a retrospectively collected dataset consisting of 61 newborns and infants with CH up to 2 years of age. Overall, 505 measurements of free thyroxine (FT4) and 510 measurements of thyrotropin or thyroid-stimulating hormone were available from patients receiving substitution treatment with levothyroxine (LT4). The second goal is to introduce a scale/location-scale normalization method to merge available FT4 measurements since 34 different postnatal age- and assay-specific laboratory reference ranges were applied. This method takes into account the change of the distribution of FT4 values over time, i.e. a transformation from right-skewed towards normality during LT4 treatment. The third goal is to develop a practical and useful PMX model for LT4 treatment to characterize FT4 measurements, which is applicable within a clinical setting. In summary, a time-dependent normalization method and a practical PMX model are presented. Since there is no on-going or planned development of new pharmacological approaches for CH, PMX based modeling and simulation can be leveraged to personalize dosing with the goal to enhance longer-term neurological outcome in children with the rare disease CH.

**Supplementary Information:**

The online version contains supplementary material available at 10.1007/s10928-021-09765-w.

## Introduction

Analysis and modeling of retrospective clinical data are important to characterize a patient cohort under treatment and to model general and individual disease progression to quantitatively describe unsolved clinical problems, e.g. in a dose- or disease severity-dependent way. Especially for rare diseases, defined as condition that affects less than 200 000 people in the US [1] or less than 1 out of 2000 people in the European Union [2], retrospective data analysis of patients diagnosed during a time window of 10 to 20 years is crucial for overcoming the limitation of the low number of patients in the general population. Rigorous retrospective analyses pave the way to successful planning of prospective studies. For rare diseases, there is often no on-going or planned development of new pharmacological approaches. Hence, personalized dosing based on pharmacometric (PMX) modeling and simulation [3, 4] is the next logical step to enhance medical treatment in patients with a rare disease. However, before general application of such PMX models, their accuracy needs to be validated in prospective controlled studies.

PMX modeling of retrospectively collected data of a rare disease originating from several decades and different centers is challenging. First, measurements of one specific laboratory parameter were performed with different commercially available laboratory assays at different centers. Second, different generations of the same laboratory assay over time in the same center are associated with varying reference ranges. Third, a majority of guidelines for standardized clinical and laboratory follow-up for a specific rare disease were developed during the last 5 to 10 years, which may result in specific clinical and laboratory investigations varying from center to center especially in older datasets.

Here we present retrospective data analysis and modeling of congenital hypothyroidism (CH) which is a rare disease (ORPHA:442) that affects 1 out of approximately 1500 to 4000 newborns [5, 6]. In patients with primary or thyroidal CH, the thyroid gland does not produce sufficient thyroid hormones leading untreated to growth failure and strongly reduced neurological outcome. Therefore, the manufactured form levothyroxine (LT4) of the thyroid hormone thyroxine (T4) is applied to treat thyroid hormone deficiency. CH is the most frequent preventable cause of mental retardation worldwide. Neurological outcome of CH patients has been strongly improved in the last 40 years by introducing systematic neonatal screening for preclinical diagnosis in the 1970s [7, 8], and by increasing the starting dose of LT4 at diagnosis from 5–10 to 10–15 mcg/kg body weight in the 1990s [9, 10]. The main aim was to aggressively correct laboratory hypothyroidism as rapidly as possible to protect thyroid-hormone dependent neurodevelopment in the newborn affected by CH, as during pregnancy, hypothyroidism of fetuses affected by CH is only partially compensated by transplacental passage of maternal thyroid hormones [11]. Recent data however revealed frequent long-lasting overdosing of patients under the recommended initial dose of 10–15 mcg/kg body weight [12, 13]. Finally, different studies showed a negative effect of long-term elevated plasma thyroxine levels during the first years of life on the intelligence quotient between 10 and 14 years [14–16]. Therefore, it is essential to develop a mathematical PMX model to characterize individual dynamics of substituted thyroxine and to further optimize and personalize LT4 treatment in the context of rapid weight gain during infancy and three disease severity levels (mild, moderate and severe CH disease according to current guidelines) [5].

This article has three goals. First, a retrospectively collected dataset consisting of n = 61 newborns and infants with CH up to 2 years of age is described. Second, due to the multi-center/-assay nature of the data, a scale/location-scale normalization method is developed to make the measured free T4 (FT4) concentrations comparable by normalization to a target reference range. For normalization, it is essential that underlying statistical assumptions of the methods are fulfilled in the dataset. Since FT4 measurements change their distribution during treatment, namely from a right-skewed distribution (scale formula) towards normality (location-scale formula), this time-dependent transition of the distribution is taken into account in the developed normalization method. Third, a mathematical model to characterize FT4 measurements and LT4 treatment has to be in a fair balance between the physiological mechanism and the capability to characterize available data. The hypothalamic-pituitary-thyroid (HPT) axis is a complex multi-loop feedback mechanism where, among many others, T4 and thyroid-stimulating hormone (TSH) control themselves to hold all thyroid hormones in a healthy equilibrium. Interestingly, mathematical modeling of the HPT axis has a long history ranging back to the 1950s [17, 18]. However, most mathematical models developed in the last decades are pretty detailed [19–21], based on animal data [22], or focus on a very specific question such as the relationship between TSH and FT4 [23, 24]. Application of such models to data collected in daily clinical routine from CH patients is usually impossible because many model parameters are not identifiable due to lack of large quantitative data. Therefore, we developed a practical PMX model describing FT4 concentration and LT4 treatment.

In summary, a PMX model for FT4 concentration and LT4 treatment based on normalized FT4 measurements is presented. The normalization method takes the transition from a right-skewed distribution towards normality during LT4 treatment into account. Such a PMX model can be applied to characterize FT4 under LT4 substitution therapy with the goal to further personalize and enhance LT4 treatment in pediatric patients with a rare disease.

## Methods

The Method section consists of six paragraphs. First, study design and retrospective data collection procedure to form the pediatric CH study population based on data from four different hospitals over the last 25 years are reported. Second, a descriptive analysis of the collected data is presented. Third, normalization procedures for laboratory reference ranges stemming from different assays are introduced. Fourth, a PMX model to characterize FT4 concentration based on remaining endogenous T4 production and exogenous LT4 administration is presented. Fifth, available covariates are discussed and finally, some remarks about applied statistics and software are presented.

### Study design

A retrospective multi-center longitudinal cohort study of consecutive pediatric patients diagnosed with primary or thyroidal CH based on elevated TSH values in neonatal screening and confirmatory laboratory testing was performed between 01/1990 and 08/2018 in Switzerland. Data from neonates and infants up to approximately 2 years of age were included if they (i) had a confirmed diagnosis of primary or thyroidal CH, (ii) were treated at the participating study centers (a) between 01/1990 to 08/2018 (University Children’s Hospital Basel, University Hospital Bern, University Children’s Hospital Zurich) or (b) between 01/1990 to 12/2013 (Children’s Hospital Eastern Switzerland, St. Gallen), (iii) had a complete dataset, including (a) clinical baseline characteristics, (b) laboratory parameters at diagnosis and/or LT4 treatment start, and (c) LT4 dose history during the complete follow-up, (iv) had ≥ 2 follow-up visits after LT4 treatment start. Patients were excluded, (i) if LT4 start dose and LT4 doses during follow-up visits were missing, (ii) in case of treatment noncompliance, or (iii) in all cases of central hypothyroidism. Data were captured standardized in the designated electronic database secuTrial® for each study visit. An ethical approval for this study (2018-01770) was obtained by the lead local Ethics Committee (Ethikkommission Nordwest- und Zentralschweiz EKNZ) and all local responsible Ethics Committees (Kantonale Ethikkommission Bern, Ethikkommission Zürich, Ethikkommission Ostschweiz EKOS). Data from patients were used pseudonymized. The study was performed in compliance with the Helsinki Declaration and Good Clinical Practice.

### Descriptive analysis of the retrospective data

In total, n = 71 pediatric patients with the rare disease CH fulfilled all inclusion criteria. Clinical records are often stored for approximately 20 years as the patients were monitored from birth until transition to an adult endocrinologist and the retention requirement is 10 years in Switzerland. In addition to the inclusion and exclusion criteria formulated above, only patients with more than 2 months of treatment were included in this analysis. Therefore, the final number of patients involved in the analysis was n = 61 (female = 70%).

**Fig. 1:**
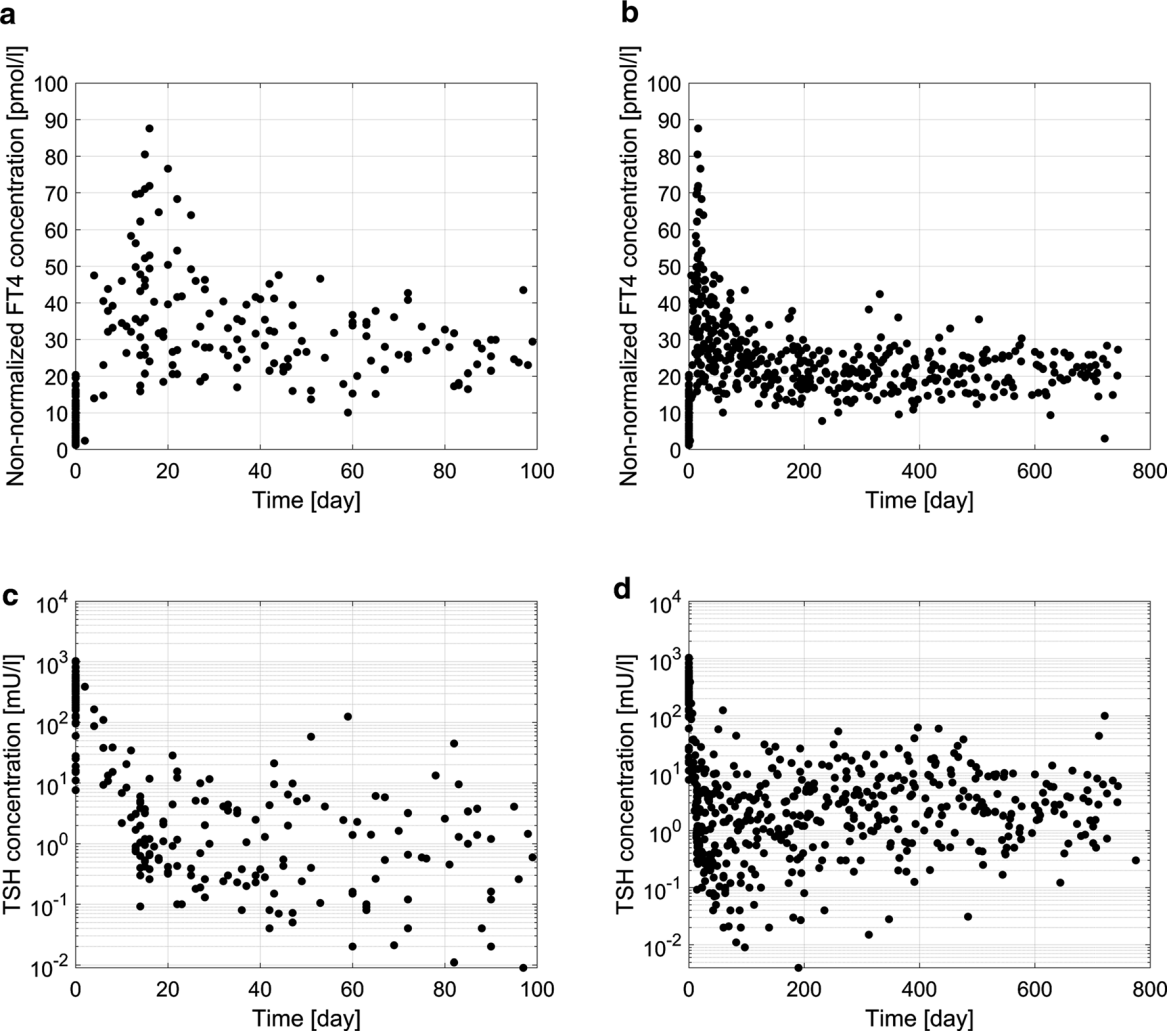
Non-normalized FT4 measurements (n = 505) are shown in panel **a** and **b**. TSH measurements (n = 510) are shown in panel **c** and **d**

Laboratory data from start of LT4 treatment $({t}_{0}$ = 0 day) were included in the analysis. Measurements of non-normalized FT4 (n = 505) and TSH (n = 510) are shown in Fig. 1. On average, 8 FT4 measurements were available per patient with minimally 4 and maximally 14 measurements. Disease severity was defined based on the first FT4 measurement at time of diagnosis, i.e. 18 patients were categorized as severe (FT4 < 5 pmol/l), 17 as moderate (FT4 ≥ 5 and < 10 pmol/l) and 21 as mild (FT4 ≥ 10 pmol/l) according to current guidelines [5]. Five patients had no initial FT4 measurement. Patient characteristics such as gestational age (GA) as well as postnatal age (PNA), weight, non-normalized FT4 and TSH concentrations, and in addition, total daily LT4 dose and LT4 dose per kg body weight all at start of treatment and last available follow-up with a FT4 measurement, are presented in Table 1.

**Table 1:** Demographic and laboratory patient characteristics at start of LT4 treatment and at last available follow-up

	Unit	Median [IQR]	[Min, Max]
Patient parameters at start of treatment			
GA^a^	week	40.5 [38.0, 41.3]	[28.3, 42.9]
PNA	day	7 [6, 9]	[3, 231]
Weight^b^	kg	3.3 [2.9, 3.8]	[0.95, 8.07]
FT4^c^	pmol/l	7.0 [3.4, 12.3]	[1.2, 20.4]
TSH^d^	mU/l	267 [146, 430]	[7.6, 1026]
LT4 dose total daily	mcg/day	25 [25, 37.5]	[10, 50]
LT4 dose per kg body weight^f^	mcg/kg/day	8.8 [6.8, 13.02]	[2.8, 28.4]
Patient parameters at last available follow-up with FT4 measurement			
PNA	day	602 [362, 708]	[98, 769]
Weight^e^	kg	11.3 [9.4, 12.7]	[4.3, 15]
FT4	pmol/l	21.0 [18.4, 25.6]	[3, 35.5]
TSH	mU/l	2.2 [0.90, 4.7]	[0.005, 100]
LT4 dose total daily	mcg/day	50 [37.5, 50]	[15, 75]
LT4 dose per kg body weight^f^	mcg/kg/day	3.8 [3.4, 4.4]	[1.8, 6.7]

^a^13 values missing; ^b^8 measurements missing; ^c^5 measurements missing; ^d^6 measurements missing; ^e^6 measurements missing; ^f^computed based on imputed values for missing weight measurements

### Normalization of FT4 concentrations with respect to different laboratory reference ranges

#### Available laboratory reference ranges of the FT4 measurements and PNA dependent target reference ranges

**Fig. 2:**
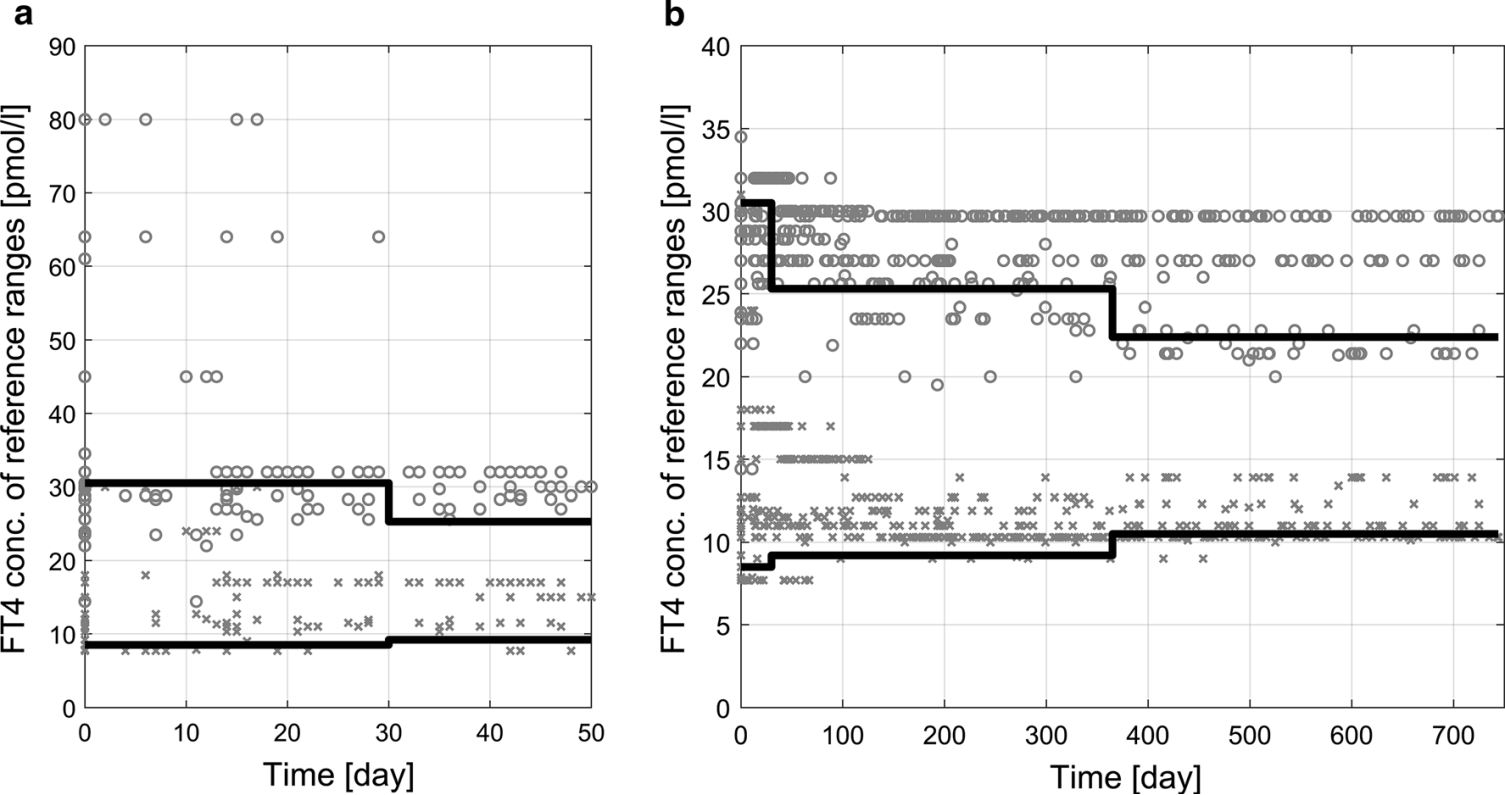
Presentation of all available laboratory reference ranges of FT4 measurements over time. Grey circles denote the upper limit and grey crosses the lower limit of each range. Black lines show the upper and lower limits of the target reference range (compare Table 2), where for simplicity in this Figure, individual PNA at start of treatment was neglected. The first 50 days are shown in panel **a**, and the total time interval is presented in panel **b**

Each of the measured FT4 values in our dataset is accompanied by a corresponding laboratory reference range. These ranges are PNA dependent but also assay- and center-related. In total, 34 different FT4 laboratory reference ranges were identified for the 505 measurements. Observed laboratory reference ranges from all FT4 measurements over time are shown in Fig. 2. We observe that during the first 30 days, few upper limits of the reference ranges are unusually large.

To merge all FT4 measurements from the four clinical centers, a normalization method was constructed based on PNA dependent target reference ranges taken from Kapelari et al. [25], see Table 2 for the 2.5 and 97.5 percentiles and Fig. 2. After normalization, the FT4 values can be treated as if they were obtained from a single standard laboratory [26].

**Table 2:** Percentiles (2.5, 50 and 97.5) of the target reference range for FT4 and TSH concentrations of neonates and infants based on postnatal age taken from [25]

	FT4 (pmol/l)			TSH (mU/l)		
Percentile	2.5	50	97.5	2.5	50	97.5
Postnatal age						
0–1 months	8.50	20.10	30.50	0.70	3.50	18.10
1–12 months	9.17	15.50	25.28	1.12	2.85	8.21
1–5 years	10.45	15.70	22.35	0.80	2.70	6.26

#### Construction of a time-dependent normalization method during treatment

**Fig. 3:**
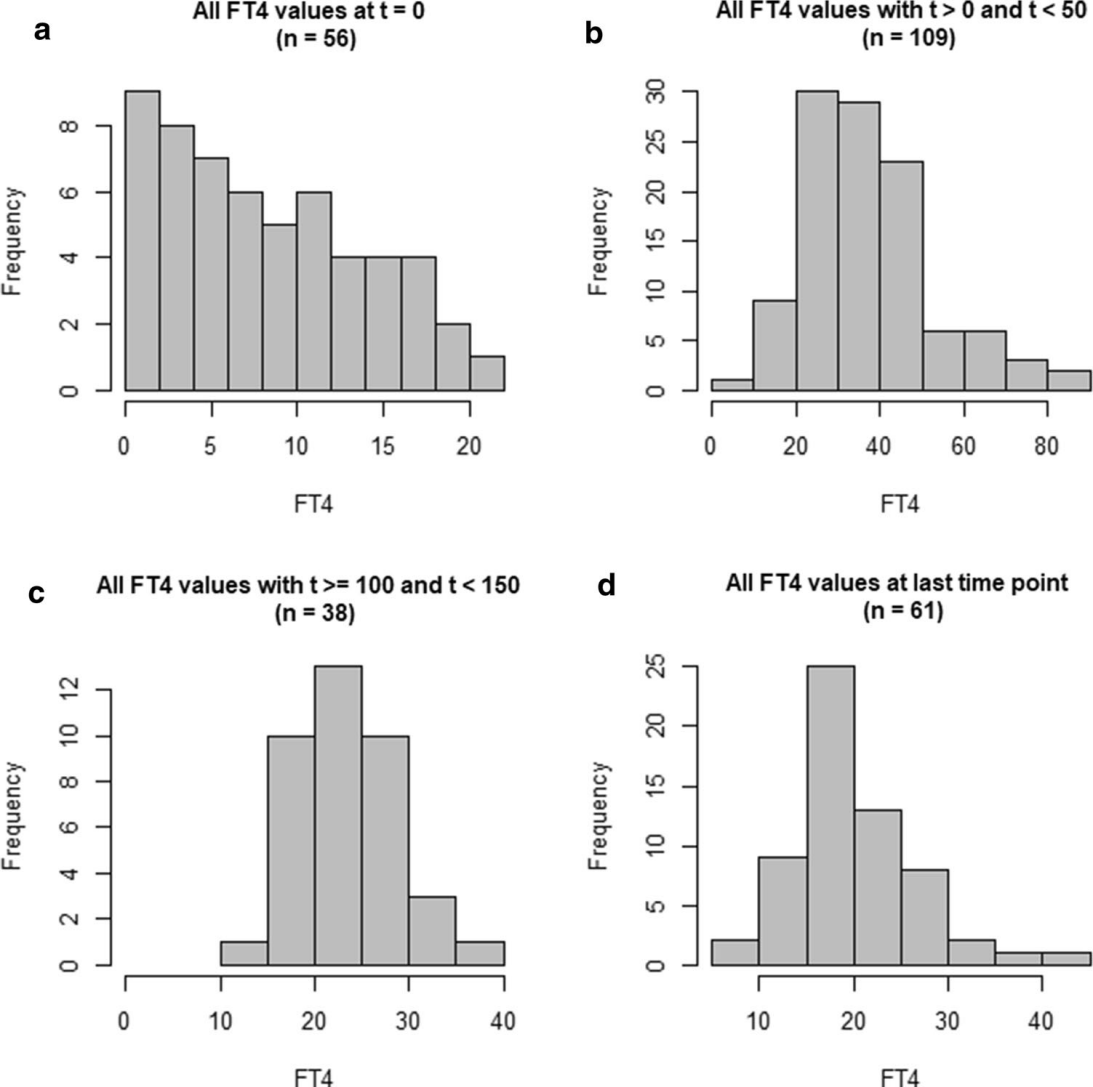
Change of distribution of the non-normalized FT4 measurements from a right-skewed distribution towards a normal distribution for increasing time of treatment. Panel **a** shows the distribution at start of treatment $t$ = 0 of all available FT4 measurements, panel **b** and **c** show the distribution for later time intervals, whereas panel **d** shows the distribution based on the individual last measurement time point

A normalization method is based on statistical assumptions which have to be verified prior to application. We observed that the distribution of FT4 measurements changes during treatment in our CH population. At time of diagnosis, non-normalized FT4 measurements follow a right-skewed distribution with several values close to zero, representing the disease severity in our cohort of severe, moderate and mild forms (Fig. 3a). However, during treatment, the distribution transforms towards normality, as shown in Fig. 3b–d for different time points and intervals. Since successfully treated patients have FT4 values in the healthy range, such a distribution is expected.

We denote with ${x}_{meas}$ the performed FT4 measurement and with ${r}_{low}$ and ${r}_{up}$ the value of the lower and upper limits of the laboratory reference range associated with this measurement. In addition, ${r}_{low}^{Std}$ and ${r}_{up}^{Std}$ denote the lower (2.5 percentile) and upper (97.5 percentile) limits of the PNA dependent target reference range taken from Table 2.

Karvanen [26] proposed and derived for right-skewed distributions a scale normalization formula to compute the normalized FT4 value ${x}_{norm}$:

1 $${x}_{norm}={x}_{meas}\cdot \frac{{r}_{up}^{Std}}{{r}_{up}}$$

For normally distributed data, it was shown that the Chuang-Stein formula [27], also called location-scale normalization formula,

2 $${x}_{norm}=\left({x}_{meas}-{r}_{low}\right)\cdot \frac{{r}_{up}^{Std}-{r}_{low}^{Std}}{{r}_{up}-{r}_{low}}+{r}_{low}^{Std}$$

is valid. For more details and justification of Eqs. (1) and (2) see [26].

Since the FT4 distribution transforms from right-skewed to normal during treatment, we propose a combination of the scale and location-scale formula. More precisely, for small times $t$ the scale formula is dominant. For increasing times $t$ up to a certain time threshold ${t}_{s}$, the scale formula transforms into a mixture of scale and location-scale formula. After the threshold ${t}_{s}$, only the location-scale formula is applied:

3 $$x_{{norm}} = \left\{ \begin{gathered} \frac{t}{{t_{s} }} \cdot \left( {\left( {x_{{meas}} - r_{{low}} } \right) \cdot \frac{{r_{{up}}^{{Std}} - r_{{low}}^{{Std}} }}{{r_{{up}} - r_{{low}} }} + r_{{low}}^{{Std}} } \right) + \left( {1 - \frac{t}{{t_{s} }}} \right) \cdot x_{{meas}} \cdot \frac{{r_{{up}}^{{Std}} }}{{r_{{up}} }}\quad for\,t \le t_{s} \hfill \\ \left( {x_{{meas}} - r_{{low}} } \right) \cdot \frac{{r_{{up}}^{{Std}} - r_{{low}}^{{Std}} }}{{r_{{up}} - r_{{low}} }} + r_{{low}}^{{Std}} \quad for\,t  > t_{s} \hfill \\ \end{gathered} \right.$$

### PMX model to characterize FT4 based on remaining endogenous T4 production and exogenous LT4 treatment

#### Part I: structural PMX model for exogenous LT4 treatment

Oral exogenous LT4 mcg/day administration is characterized with the input function $In({t}_{j},dos{e}_{j},F)$, where ${t}_{j}$ is the dosing time point, $dos{e}_{j}$ the total daily LT4 dose mcg/day and $F$ the bioavailability. The dose was converted with factor 1.29 to nmol/day (molecular weight of thyroxine 776.9 g/mol). The absorption compartment ${A}_{B}$ nmol reads

4 $$\frac{d}{dt}A_{B} = In\left( {t_{j} ,dose_{j} ,F} \right) - k_{a} \cdot A_{B} \, , \quad A_{B} \left( 0 \right) = 0$$

with absorption rate ${k}_{a}$.

#### Part II: structural PMX model for endogenous T4 production

We apply a typical one-compartment pharmacokinetic model with linear first-order elimination and further assume that a remaining endogenous T4 production is present, described with the zero-order production rate ${k}_{endo}$ nmol/day

5 $$\frac{d}{dt}A_{C} = k_{a} \cdot A_{B} + k_{endo} - k_{el} \cdot A_{c} \, , \quad A_{C} \left( 0 \right) = \frac{{k_{endo} }}{{k_{el} }}$$

where ${A}_{C}$ characterizes the central compartment with the amount of T4 nmol. Linear elimination is described with the first-order elimination rate ${k}_{el}$ 1/day and the initial condition assumes that the endogenous T4 production is in an equilibrium prior to treatment. This assumption might not be fully correct at start of treatment since the neonate may still slightly benefit from transplacental passage of maternal hormones until birth. However, after stopping the treatment the amount of T4 will always return to the equilibrium $\frac{{k}_{endo}}{{k}_{el}}$ given in Eq. (5) which is consistent with the assumption that the disease cannot be cured.

#### Part III: structural PMX model with body weight development

The FT4 concentration is derived by transforming the T4 unit nmol to pmol and assuming that the FT4 concentration corresponds to 0.03% of T4 [28]. Therefore, FT4 concentration pmol/l reads

6 $${C}_{FT4}=0.3\cdot \frac{{A}_{C}}{V(W)}$$

where $V$ l is the volume of distribution depending on current body weight kg $W(t)$:

7 $$V\left(W\right)={f}_{V}\cdot {\left(\frac{W\left(t\right)}{{W}_{Ref}}\right)}^{{\beta }_{W}}$$

where ${f}_{V}$ l is a multiplicative factor relating body weight with volume of distribution, ${W}_{Ref}$ is the median of body weight of the underlying population, and ${\beta }_{W}$ the power exponent. Obviously, if the treatment stops, FT4 concentration will return to an equilibrium which depends on the current body weight.

#### Interpolation of body weight and TSH over time

In total, n = 474 measurements of body weight $W$ were available. Daily values for body weight and TSH were imputed by non-linear interpolation.

#### Covariate modeling

Categorical (not time-varying) covariates were implemented and tested with the default setting from The Monolix Suite 2020R1 (Lixoft, Orsay, France). Continuous covariates were tested at model parameters with the power model

$${\theta }_{i}={\theta }_{pop}\cdot {\left(\frac{Co{v}_{i}}{Co{v}_{Ref}}\right)}^{\beta }$$

where ${\theta }_{i}$ is the individual model parameter, ${\theta }_{pop}$ is the population model parameter (typical value), $Co{v}_{i}$ is the individual (possibly time-varying) covariate, $Co{v}_{Ref}$ is the reference covariate value and $\beta$ is the exponent describing the covariate effect.

### Covariate selection and testing

Covariates to be tested were selected based on completeness, possible correlations among each other and clinical plausibility.

GA was not available for all patients, however strongly correlates with birth weight, and was therefore not tested. PNA at start of treatment (continuous) and sex (categorical) were included in covariate testing.

Body weight over time (time-varying) with the interpolated values for missing measurements was already incorporated in the model to compute the volume of distribution, compare Eqs. (6) and (7). Body weight at start of treatment (continuous) was not selected for testing because 8 measurements were missing and body weight over time was already included.

TSH effects over time on the endogenous production rate were tested with two different groupings. In the first group, TSH values were categorized as follows: category 1 with TSH < 3 mU/l (milliunits per liter) and category 2 with TSH ≥ 3 mU/l. The value 3 mU/l was selected since it corresponds to the average of the median target reference range in our study population**,** compare Table 2. In the second group, TSH values were split into three categories: category 1 TSH < 1 mU/l, category 2 TSH ≥ 1 and TSH < 10 mU/l, and category 3 TSH ≥ 10 mU/l. Chosen values roughly correspond to the average of the lower and upper limits of the target reference range, compare Table 2. Although it is obvious that a relationship between TSH at start of treatment (continuous) and FT4 at start of treatment exists, TSH at start of treatment was not selected because 6 measurements were missing.

### Statistical data presentation and applied software

All laboratory and demographic values are reported as median together with the interquartile range (IQR) [25%, 75%]. Descriptive statistical analysis was performed in R 3.6.0 (R core team, Vienna, Austria) and hypothesis testing was executed with the Student’s t-test for normally distributed values and with the Wilcox rank test for non-normally distributed values. Non-linear mixed-effects modeling was performed in The Monolix Suite 2020R1 (Lixoft, Orsay, France). A-posteriori data visualization was implemented in R or Matlab 2020a (MathWorks, Natick, MA, USA).

## Results

The Results section consists of five paragraphs. First, the non-normalized and normalized measurements are compared. Second, model parameter estimates are presented. Third, a TSH feedback effect on FT4 is tested. Fourth, the final PMX model for CH is presented. Fifth, additional tests are performed.

### Comparison of non-normalized and normalized measurements

Transition from a right-skewed towards a normal distribution was expected at time threshold ${t}_{s}$ = 150 day, compare Fig. 3. After application of the proposed scale/location-scale formula Eq. (3), we observe that normalized FT4 values are slightly but not significantly lower than non-normalized measurements. Comparison for start of treatment (${t}_{0}$ = 0), additional time intervals 0 $<t<$ 50, 100 $\le t<$ 150, and at $t$ = last available time point are presented in Table 3.

**Table 3:** Comparison of non-normalized and normalized FT4 values for different time points and time intervals

Time points/intervals (day)	Non-normalized FT4 (pmol/l) Median [IQR]	Normalized FT4 (pmol/l) Median [IQR]	Significance
Start of treatment (${t}_{0}$ = 0)	7.0 [3.4, 12.3]	5.2 [2.4, 10.4]	N.s.^a^
0 $<t<$ 50	33.6 [26.3, 45.2]	31.7 [22.6, 43.2]	N.s.^a^
100 $\le t<$ 150	23.4 [19.5, 26.3]	20.6 [18.0, 24.1]	N.s.^a^
Last available time point	21.0 [18.4, 25.6]	18.0 [16.0, 22.7]	N.s.^a^

^a^Not significant

We emphasize that the application of the Chuang-Stein (location-scale) formula alone resulted in 29 negative values for FT4 at start of treatment. This was expected since the normal assumption was violated (compare Fig. 3a) and laboratory reference ranges were quite large compared to the actual FT4 value.

TSH over time is a highly variable and sensitive marker with absolute values ranging from the limit of quantification up to 1026 mU/l in our study population, compare Fig. 1b. An investigation of the distributions of TSH at specific time points showed mostly right-skewed behavior with no clear tendency towards normal (data not shown). In total, 43 TSH measurements were not accompanied by laboratory reference ranges (incomplete or missing) and therefore, TSH was not normalized.

### PMX model parameter estimation based on normalized FT4 values

LT4 is variably absorbed and bioavailability is reported between 40 to 80% (https://go.drugbank.com/drugs/DB00451). We fixed the bioavailability to $F$ = 0.6 in Eq. (4). The model Eqs. (4)–(7) consists of the structural model parameters ${k}_{a}$, ${k}_{endo}$, ${f}_{V}$ and ${k}_{el}$. The absorption rate ${k}_{a}$ was fixed to 20 1/day, realizing a maximal FT4 peak at 2 h [29], and had no inter-individual variability (IIV). The endogenous production rate ${k}_{endo}$, the factor ${f}_{V}$ relating body weight with volume of distribution, as well as the power exponent ${\beta }_{W}$ were estimated with a log-normal distribution. Based on available data, the elimination rate ${k}_{el}$ is difficult to estimate. Since T4 half-life in plasma is on average 7 days [29], ${k}_{el}$ was fixed to 0.1 1/day. Allowing IIV for ${k}_{el}$ resulted in a high shrinkage based on the individual conditional mode estimations and a small standard deviation of the random effects. Hence, IIV on this parameter was omitted. We remark that a formulation of the model in terms of clearance with a weight-based allometric scaling approach was omitted because of (i) insufficient FT4 data to reasonably apply such an approach, (ii) the lack of information regarding maturation effect on the half-life in literature, and (iii) the rather small weight range. Data fitting was performed with a proportional residual error model.

PNA at start of treatment showed a significant but weak effect on the endogenous production rate ${k}_{endo}$. However, the PNA effect was exclusively driven by 4 patients with PNAs larger than 50 days. Without these 4 patients, no significant effect was available. Therefore, the PNA effect was not included in the final model. Sex had no effect on any model parameter.

### Test for TSH feedback effects on FT4

Low FT4 (or T4) values cause increased TSH levels which in turn will try to stimulate the FT4 (or T4) production. Therefore, we tested a TSH feedback on the endogenous T4 production rate ${k}_{endo}$. More precisely, ${k}_{endo}$ is modeled as a function of TSH and changes over time, denoted by ${k}_{endo,Cov}$. Hence, ${k}_{endo}$ is now substituted by ${k}_{endo,Cov}$ in Eq. (5). First, the previously defined two time-varying categorical TSH groups were tested. For the first grouping we applied

$${k}_{endo,Cov}=\left\{\begin{array}{l}{k}_{endo} \,\qquad \, \, \mathrm{for\,category} \, 1 \\ {k}_{endo}+{\beta }_{1}\, \mathrm{for\,category} \, 2\end{array}\right.$$

and for the second grouping:

$${k}_{endo,Cov}=\left\{\begin{array}{l}{k}_{endo} \qquad \, \, \mathrm{\, for\,category} \, 1\\ \begin{array}{c} \\ {k}_{endo}+{\beta }_{1} \mathrm{\, for\,category} \, 2\end{array}\\ \begin{array}{c} \\ {k}_{endo}+{\beta }_{2} \mathrm{\, for\,category} \, 3\end{array}\end{array}\right.$$

Second, the effect of actual TSH measurements on ${k}_{endo}$ were tested with

$${k}_{endo,Cov}={k}_{endo}\cdot f\left(TSH\right)$$

and

$${k}_{endo,Cov}={k}_{endo}+{\beta }_{3}\cdot f\left(TSH\right)$$

The function $f\left(TSH\right)$ was either (i) $f\left(TSH\right)=TSH$, (ii) $f\left(TSH\right)={\left(\frac{TSH}{TS{H}_{Ref}}\right)}^{\beta }$, (iii) $f\left(TSH\right)=\mathrm{log}\left(TSH\right)$, or (iv) $f\left(TSH\right)={\left(\frac{\mathrm{log}\left(TSH\right)}{\mathrm{log}\left(TS{H}_{Ref}\right)}\right)}^{\beta }$.

Based on the available data, it was difficult to observe an improvement in data fitting by including a TSH feedback on the endogenous production rate. With a combined assessment of typical data fitting criteria such as goodness-of-fit plots, reduction of the objective function, reduction of variability in random effects, and changes in standard error, no overall improvement could be concluded. Therefore, TSH feedback effect was not included in the model.

### Final PMX model for CH based on normalized FT4 values

**Fig. 4:**
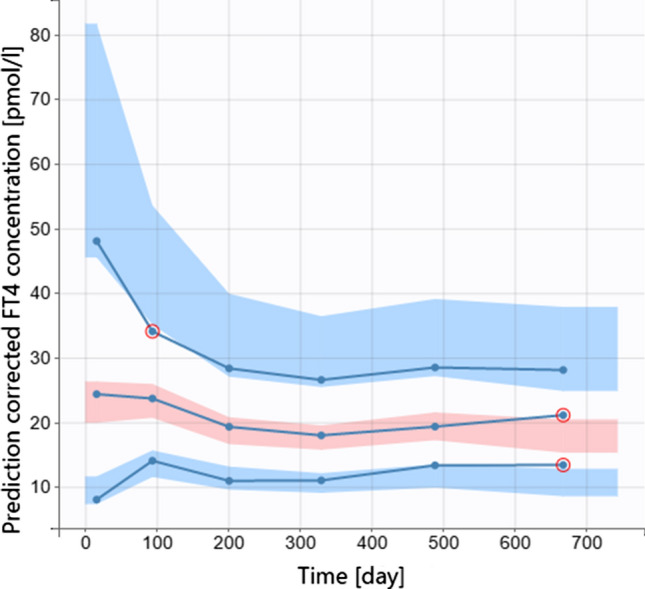
Visual predictive check based on normalized FT4 measurements modeled with Eqs. (4)–(7)

The final model consists of Eqs. (4)–(7) without any additional covariate effects. Final model parameter estimates are shown in Table 4. Visual predictive check is presented in Fig. 4. Other goodness-of-fit plots and individual profiles are available in the supplemental material.

**Table 4:** Population estimates (typical values), standard deviation of the random effects and additional parameters obtained from fitting normalized FT4 data with the final PMX model Eqs. (4)–(7)

Parameter name	Description	Unit	Estimate (r.s.e.^a^)
Population estimates (fixed effects)			
${k}_{a}$	Absorption rate	1/day	20 fix
${k}_{el}$	Elimination rate	1/day	0.1 fix
${f}_{V}$	Multiplicative factor	l	4.96 (3.7)
${\beta }_{W}$	Power exponent	–	0.753 (6.6)
${k}_{endo}$	Endogenous production rate	nmol/day	3.66 (15.7)
Standard deviation of the random effects			
${\omega }_{{f}_{V}}$			0.249 (11.9)
${\omega }_{{\beta }_{W}}$			0.404 (15.8)
${\omega }_{{k}_{endo}}$			1.12 (11.1)
Additional parameters and values			
Prop. residual error			0.228 (4.2)
− 2LL value			3268

^a^Relative standard error

### Additional tests

In the following, an additional test was performed to further verify the final PMX model Eqs. (4)-(7).

#### Comparison of results with parameter estimates from non-normalized data

With the final model Eqs. (4)–(7), the non-normalized FT4 data were fitted and individual parameter values were compared. The estimated individual parameter values for the endogenous production rate ${k}_{endo}$ for non-normalized FT4 values were significantly higher (p < 0.005) than for normalized values. Comparison of estimated individual parameter values for ${f}_{V}$ and ${\beta }_{W}$ is more complicated since the volume of distribution depends on both parameters as given in Eq. (7). Hence, for any individual we have to consider the pair $({f}_{V}, {\beta }_{W})$ and compare the resulting volume of distribution obtained from non-normalized FT4 values to the resulting volume of distribution obtained from normalized FT4 values. We restrict the comparison to the volume of distribution at baseline and the volume of distribution for median body weight. In both situations, we observe that the volume of distribution for non-normalized FT4 values is significantly lower (p < 0.005) than for normalized data. Hence, these comparisons coincide with the fact that normalized FT4 data were on average lower than non-normalized FT4 data, compare Table 3.

## Discussion

In this section we discuss the key findings in the context of the three main goals of this research project: (i) Presentation of challenges associated with a retrospectively collected dataset including long-term follow-up of 61 newborns and infants with a rare disease (CH). (ii) Derivation of a time-dependent scale / location-scale normalization method to account for the multi-center/-assay nature of the data. (iii) Development of a practical PMX model describing FT4 concentration and LT4 treatment.

The first goal of this article was to describe retrospectively collected multi-center data from newborns and infants with CH up to 2 years. Clinical and laboratory records from children with a rare disease are stored for approximately 20 years as such patients were followed from birth until transition to an adult endocrinologist and the retention requirement is 10 years in Switzerland. This allowed for collecting data from patients ranging back to 1995 resulting in n = 61 newborns with CH after application of inclusion and exclusion criteria. Assuming approximately 80,000 live births per year in Switzerland in the last decades (https://www.bfs.admin.ch) leads to the total number of 1.8 million live births from 1995 to 2018. The incident rate of CH in Switzerland is 1:3500 (https://www.neoscreening.ch) resulting in approximately 515 newborns with CH between 1995 and 2018. Hence, our dataset roughly consists of 12% of all newborns with CH in Switzerland from 1995 to 2018. The dataset revealed the major problem of strongly variable FT4 laboratory reference ranges especially in the first weeks of life. There are several reasons for this. First, some reference ranges were simply adult references not taking into account the higher normative values in the neonates and young infants, while other reference ranges were adapted to the neonatal and infant age group. Second, during long-term observation, laboratory assays and consequently reference ranges changed for the same parameter within a center. Third, different laboratory methods were used at different centers and changed at different time points. Harmonization and normalization of the clinical dataset was an important part of the work before any modeling of the disease and the therapy could have been done. While prospectively planned studies allow avoiding such technical hurdles, in rare diseases with a limited number of patients at a single center and even in a region or country, the retrospective approach has the advantage to establish datasets for generating hypotheses and modeling in parallel to the realization of a prospective study. In summary, retrospective data analyses are feasible even in the context of, as in our case, 34 different laboratory reference ranges, if normative data standardization is implemented.

The second goal was to develop a normalization method. However, before developing such a method, we have to discuss the well-known question of whether normalization is indeed necessary. The answer depends on the particular situation. On one hand, in drug development, different reference ranges can usually be avoided by applying standardized assays in the same laboratories right from the beginning of the study. If this standardization is not possible, it is suggested to ignore laboratory differences and “*the analyst must accept the data as is*” [30] or consider the application of normalization methods “*as the last resort*” [31]. On the other hand, our multi-assay/-center dependent nature of the measurements, as previously explained, calls for a normalization method to reasonably merge available data. The Chuang-Stein formula is a linear approach to normalize data with different reference ranges**.** Hence, normally distributed data will remain normally distributed after application of the Chuang-Stein formula. However, the Chuang-Stein formula can produce negative normalized values in specific situations. This issue was reported by Chuang-Stein [31] and was also observed when applying the Chuang-Stein formula to our FT4 measurements. Another issue is the assumption of normality itself which is not fulfilled at start of treatment as shown for our FT4 measurements. For such right-skewed distributed data, Karvanen [26] derived a scale formula for normalization. Therefore, we developed a time-dependent normalization method that is based on the scale formula in the beginning of treatment and transitions to the location-scale formula for normally distributed data when the FT4 values are in the healthy range.

The third goal was to develop a practical PMX model for LT4 treatment to characterize FT4 measurements. The major aim was that the PMX model can be applied in a clinical setting. Therefore, the PMX model has to be in a fair balance between the physiological mechanism and the capability to be applied to routinely collected clinical data. Already in the 1950s, Danziger et al. [18] published two systems of non-linear differential equations that describe the thyroid-pituitary homeostatic mechanism and performed a mathematical analysis of the model structures. Notable from our perspective is the work by Mak et al. [19] who developed a model for hypothyroidism consisting of 6 compartments describing T3 and T4 concentration in plasma, extravascular tissue and general tissue, and 17 model parameters. Other models taking complex details into account to characterize interactions in the HPT axis were developed by Leow [32], Degon et al. [33], or Eisenberg et al. [21]. Mukhopadhya et al. [34] and Berberich et al. [35] even applied delay differential equations [36] to capture existing time delays in the interactions. Remarkable is the systems pharmacology model by Ekerot et al. [22] based on pharmacokinetic/pharmacodynamic concepts to describe the impact of thyroperoxidase inhibition in dogs where model parameters are estimated with non-linear mixed-effects modeling. On one hand, all of these models offer great insights into the detailed mechanism of the HPT axis but they are on the other hand difficult to apply to clinical data due to their complexity. Development of an applicable and predictive mathematical model for clinical practice is a tradeoff between available data, prior knowledge from literature, complexity of the underlying physiological and pathophysiological mechanisms, and, most importantly, the capability to address clinically relevant research questions. The final structure of our developed PMX model for FT4 measurements and LT4 treatment was a one-compartment model with absorption and an additional zero-order endogenous production rate. The endogenous production rate of T4 allows testing for TSH feedback effects. More precisely, low FT4 levels cause increased TSH levels which in turn should stimulate FT4 production. TSH is a highly variable biomarker for thyroid functionality. However, it is unclear how strong the TSH feedback effect is on the FT4 production in CH patients. We tested different mathematical terms to estimate the effect of TSH concentration on the endogenous production rate of T4 but could not identify a significant effect based on (i) estimated model parameters, (ii) reduction of objective function value, and (iii) diagnostic plots. The following three reasons may explain these unexpected results. First, the cause of the disease is unresponsiveness or reduced responsiveness of the thyroid gland to produce FT4 to even high levels of TSH as seen at the FT4 and TSH values at diagnosis. Thus, the expected stimulatory effect of TSH is not or clearly less efficient in patients with CH than in healthy individuals. Second, only 52 of 505 measurements represent pairs of TSH and FT4 before start of LT4 treatment and an effect would only be found gradually in the moderate to mild forms further reducing the numbers. Third, after initiation of substitutive treatment with LT4, TSH is falling back into the target reference range as the result of a normally functioning negative feedback loop to exogenously administered LT4 on TSH synthesis and secretion. Further, TSH remains in the target reference range during long-term treatment, if the dose is ideally adapted to the needs of the growing child. Lack of modeling a feedback from TSH on FT4 should not be overestimated since it makes the developed PMX model even more applicable to the clinical setting, where TSH measurements might be missing.

In summary, this research article discussed challenges in analyzing retrospective data from pediatric patients with a rare disease and presented a practical and clinically useful PMX model that characterizes FT4 concentration under LT4 treatment in newborns and infants with CH.

## Supplementary Information

Below is the link to the electronic supplementary material.

Supplementary file1 (DOCX 192 KB)
