# Supplementary material for: Modeling of levothyroxine in newborns and infants with congenital hypothyroidism: challenges and opportunities of a rare disease multi-center study
Source: J Pharmacokinet Pharmacodyn. 2021 Jun 11;48(5):711–23. doi: 10.1007/s10928-021-09765-w (PMC8405503; doi:10.1007/s10928-021-09765-w)
Supplement: Supplementary file 1 — Supplementary file1 (DOCX 192 KB) [file 10928_2021_9765_MOESM1_ESM.docx]

Version: April 12, 2021

**Supplemental material**

**for**

**Modeling of levothyroxine in newborns and infants with congenital hypothyroidism: challenges and opportunities of a rare disease multi-center study**

**Content:**

Figure S1: Goodness-of-fit plots

Figure S2: Twelve (out of 61) individual profiles

**a b**


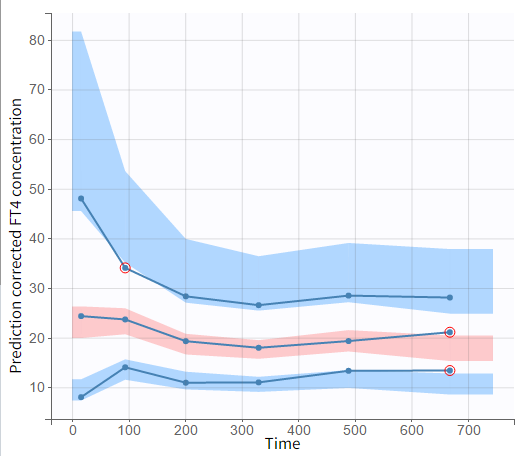


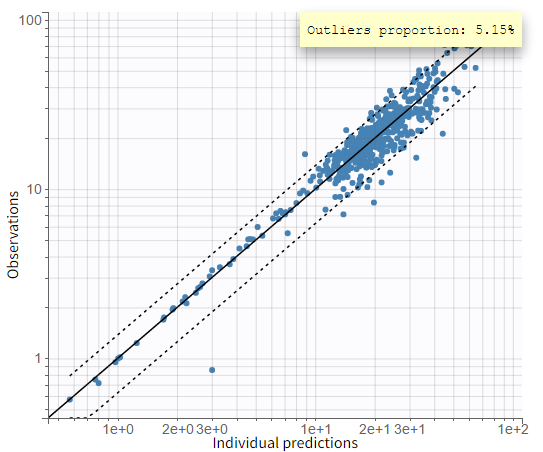


**c d**


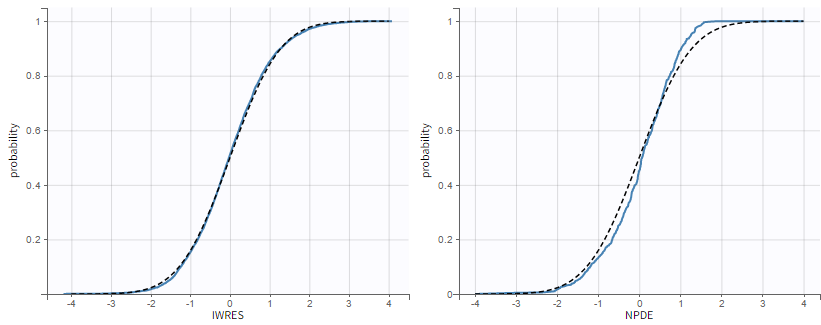


**Figure S1:** Prediction vs. observation is presented in panel **a**, where the orange curve is the spline. Visual predictive check with 10^th^, 50^th^ and 90^th^ percentiles is shown in panel **b**. In addition, individual weighted residuals (IWRES) (panel **c**) and the normalized prediction distribution error (NPDE) (panel **d**), each vs. the Gaussian cumulative distribution function, are shown.


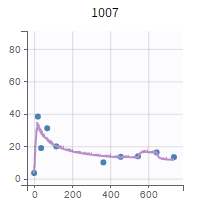

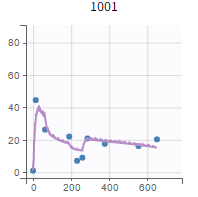


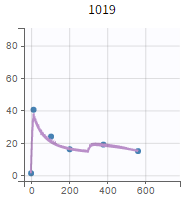

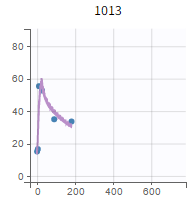


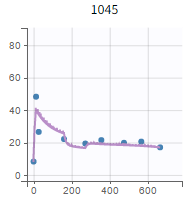

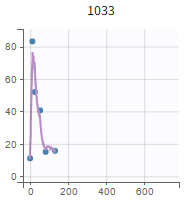

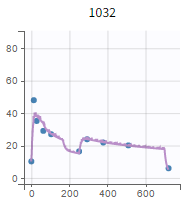

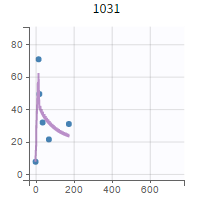


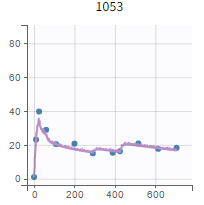

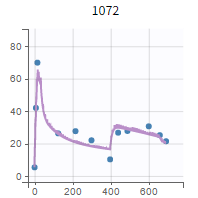

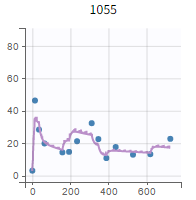

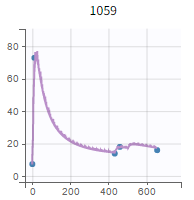


**Figure S2**: Twelve (out of 61) individual FT4 concentration-time profiles are shown. These profiles (i) reveal the complexity of the FT4 data, (ii) show the extreme high FT4 concentrations shortly after start of treatment, and (iii) demonstrate the long-term dynamic of FT4.
